# Cancer Cure

**Published:** 1872-05

**Authors:** 


### CANCER CURE.

The newspapers have extensively circulated a statement that mountain tea, drank freely and used as a poultice, had a remarkably curative effect in a case of cancer, but no instructions were given as to the nature of the plant, or where it could be obtained, nor was any direction afforded by which any practical use could be made of the information given in such an unsatisfactory and tantalizing manner.

This mountain tea is a kind of evergreen, growing in light, sandy soils, enriched by decaying leaves. It loves damp and shady places, as under trees, running on the ground, covering it from sight, and has a deep green foliage, with a perennial, creeping, yellowish root; the leaves are wedge-shaped, and smooth; the edges are saw-like, of a shining deep green color on top, a lighter green under; the flowers stand on nodding stems. This beautiful and cooling looking plant grows clear across the American Continent, and flowers in June and July. There is virtue in all the plant; the leaves are for sale in the drug-stores, and are of a sweetish-bitter taste; the root is more pungent. The virtues of the plant are obtained by boiling in water, or steeping in alcohol.

The famous Doctor Mitchell first brought it to public notice, in a paper read in New York city in 1803, on his graduation. It promotes urination, gives tone to the stomach, as other bitters do, and has astringent qualities like common green tea, or white oak bark, and like them is good for healing ulcers, scrofulous sores, and the like. It has not hitherto been supposed to have any cancer-curing virtues; but as there are many troublesome and old sores that seem to be cancer to the uneducated and inexperienced, but which are really not, it may readily be supposed that under such misapprehension an uncultivated person might become very enthusiastic under a mistaken idea; and the very "cancer" which was cured by the application of a poultice of mountain tea to the nose, might have been healed as well by the "ooze" of white oak bark, as the country people are fond of expressing themselves.

Either boil the leaves, making of them a pretty strong tea, and drink a pint every twenty-four hours; it gives tone to the stomach, and thus makes digestion more vigorous, and makes a more healthful, purer blood, and to that extent cuts off the supply of yellow matter which sores throw out; or a syrup may be made thus: four ounces of the leaves, bruised, to remain in half a pint of cold water, that is, sixteen tablespoonfuls, for thirty-six hours; put this in a bag, and pour cold water into this bag until a pint has leaked through the bottom; then warm or simmer it by the fire until it is reduced to half a pint, then stir into it twelve ounces of sugar until dissolved; then give it to a pig, or take a tablespoonful or two three times a day until you get a cancer; then poultice this cancer with mountain tea leaves until you are cured of the almost universal dread among women, of cancer. Who ever knew a woman who was not afraid of cancer?

The botanist will know the plant by the name of Chimaphila umbellati; sex system, Decandria monogynia; natural order, Pyrolaceæ; calyx, five toothed; petals, five; style, very short; stigma, annular and orbicular, with a five lobed disk; filaments, stipitate; stype, discoid ciliate; capsules, five celled, opening from five summits, margins unconnected. Now if after the trouble we have taken to spell out all these hard names, any herbman can't easily tell the mountain tea from a barn door, it must be given up as a bad job. Common country people, who don't know what botany is, will recognize "mountain tea" as an old acquaintance under the name of

PIPSISSEMA.

Others may know it by the name of partridge-berry, deer-berry, tea-berry, or

WINTER-GREEN.

Perhaps now that the reader knows all about the cancer-curing mountain tea, and that a ton of it can be had anywhere upon this broad continent for the gathering, he won't think it worth while to try it to cure cancer or anything else.

